# Telemedicine transforming stroke care in Piauí: an analysis of a state-wide initiative's first year

**DOI:** 10.1055/s-0045-1807719

**Published:** 2025-06-01

**Authors:** Irapuá Ferreira Ricarte, Ícaro Araújo de Sousa, Romilto Costa Pacheco Neto, Arthur de Oliveira Veras, Camila Victória da Silva, Cristina Calmon de Araújo Mascarenhas, Matheus Felipe da Silva, Gisele Sampaio Silva, Octávio Marques Pontes-Neto

**Affiliations:** 1 Universidade Federal de São Paulo, Escola Paulista de Medicina, Departamento de Neurologia e Neurocirurgia, São Paulo SP, Brazil.; 2 Centro Universitário UNINOVAFAPI, Departamento de Ciências da Saúde, Teresina PI, Brazil.; 3 Universidade de São Paulo, Faculdade de Medicina de Ribeirão Preto, Departamento de Neurociências e Ciências do Comportamento, Ribeirão Preto SP, Brazil.; 4 Universidade de São Paulo, Faculdade de Medicina de Ribeirão Preto, Departamento de Imagens Médicas, Hematologia e Oncologia Clínica, Ribeirão Preto SP, Brazil.

**Keywords:** Stroke, Telemedicine, Thrombolytic Therapy, Healthcare Disparities, Brazil

## Abstract

**Background:**

Prior to October 2022, stroke patients in Piaui lacked access to both stroke unit care and thrombolytic treatment, until the implementation of a telemedicine-based stroke care program.

**Objective:**

To conduct a descriptive analysis of patients suspected of stroke who received telemedicine-based care in Piauí during the first year of the program's implementation.

**Methods:**

We conducted a retrospective analysis of 868 patients treated from October 2022 to September 2023 under the state-wide stroke care program, which includes 6 Stroke Treatment Centers distributed around the state. Utilizing data from the JOIN telemedicine platform, the analysis focused on patient demographics, stroke types, treatment timelines, and outcomes of thrombolytic treatment.

**Results:**

The cohort included 868 patients, with a mean age of 67.2 years, out of whom 55.5% were male. Chronic hypertension was reported in 57.8% and diabetes in 22.5% of the patients. The average onset-to-door time was 3.3 hours, door-to-computed tomography time was 23 minutes, and door-to-needle time averaged 67 minutes. Thrombolysis was administered to 15.6% of all stroke cases, with 31.8% among those being ischemic strokes. Among patients who arrived within the thrombolytic time window, the rate was 27.3%.

**Conclusion:**

The introduction of a telemedicine-based stroke care program in Piauí has significantly enhanced access to essential stroke treatments, demonstrating its effectiveness in overcoming healthcare disparities. The initiative's success underscores the potential of telemedicine as a scalable model for improving stroke care in regions with limited healthcare resources. Future efforts will focus on reducing treatment times and expanding stroke care infrastructure to ensure comprehensive patient care.

## INTRODUCTION

Stroke has risen to become Brazil's leading cause of death as of 2022, with its survivors often facing severe physical and cognitive challenges that significantly reduce their quality of life and present a substantial economic burden. 1 The healthcare system in Brazil exhibits a stark disparity in the availability of stroke care, with affluent regions having access to advanced reperfusion therapies. In contrast, public hospitals, the primary healthcare providers for most of the population, often do not have access to essential thrombolytic treatments and standardized care protocols. 2 This gap is most acutely felt in Piauí, one of the poorest states in Northeast Brazil, and a region marked by pronounced socioeconomic challenges.

Data from the Project for the Evaluation of the Health System's Performance (PROADESS) places Piauí at the epicenter of a critical health crisis, with the highest cerebrovascular disease mortality rate in Brazil at 70.2 deaths per 100,000 inhabitants, significantly exceeding the national average of 44.9. This alarming rate highlights the imperative need for a comprehensive stroke treatment program launched statewide in 2022, 3 aimed at confronting these severe health challenges. Central to this program is the telestroke initiative, utilizing telemedicine to connect patients in remote areas with stroke specialists. This innovative strategy is expected to reduce treatment delays and improve patient outcomes, serving as a crucial solution in Piauí, which suffers from a pronounced shortage of neurologists in rural areas and holds the country's highest stroke mortality rate. Telemedicine stands as a critical solution to navigate the challenges of geographical and resource limitations, accelerating diagnosis and the delivery of care and enabling specialists in major healthcare centers to extend their support to the regions most affected. 4 5 6 7

Our study aims to conduct a descriptive analysis of patients suspected of stroke who received telemedicine-based care in Piauí during the first year of the program's implementation. We seek to assess the impact of telemedicine on improving stroke care in the region and to provide a comprehensive overview of the telestroke initiative's operational framework. Significantly, this program's implementation in Piauí represents a crucial step toward reducing healthcare disparities in stroke care, as no thrombolytic treatments were previously recorded in the state's public healthcare facilities. This initiative promises to transform the approach to stroke treatment in regions burdened by limited healthcare resources, setting a precedent for similar healthcare environments.

## METHODS

The present retrospective study evaluates the inaugural year of the statewide integrated stroke care program in Piauí, Brazil, initiated in October 2022. The program's core objective is to deliver prompt thrombolytic treatment within the 4.5-hour window following a stroke, optimizing patient outcomes by reducing door-to-needle times and associated morbidity and mortality.

### Study protocol

The current study encompassed all patients treated under the Piauí state stroke care pathway from October 2022 to September 2023. A retrospective analysis of patient records was conducted to collect demographic and clinical information. Data collected included demographics, stroke risk factors, stroke subtypes based on computed tomography (CT) (ischemic or hemorrhagic stroke) and transient ischemic attack (TIA), patient disposition during hospital admission (intensive care unit, stroke unit, regular ward, or emergency room), frequency of treatment with thrombolysis or thrombectomy. The analysis included evaluating stroke risk factors and characteristics of the stroke (including stroke type and baseline National Institutes of Health Stroke Scale [NIHSS] scores), and details regarding the timing and approaches of acute management (such as time of hospital arrival, door-to-CT times, door-to-needle times, and the type of recanalization therapy administered).

﻿The subtypes of stroke were defined using universally accepted criteria: ischemic stroke and intracerebral hemorrhage based on clinical and neuroimaging findings (CT or magnetic resonance imaging [MRI]). 8 Transient ischemic attack was defined as a transient episode of neurological dysfunction caused by focal brain, spinal cord, or retinal ischemia without acute infarction, with symptoms usually lasting < 24 hours. 9 10 Risk factors were considered if described on the patient's chart.

### Data collection

Patient data was sourced from the JOIN telemedicine platform and used to manage patients with suspected stroke. This platform maintains a comprehensive epidemiological and clinical data archive for patients treated within the Piauí stroke care program. Data retrieval was conducted remotely via the JOIN application, adhering to strict confidentiality standards through robust anonymization protocols inherent to the system.

### Regionalization and facility deployment

**Figure 1.**
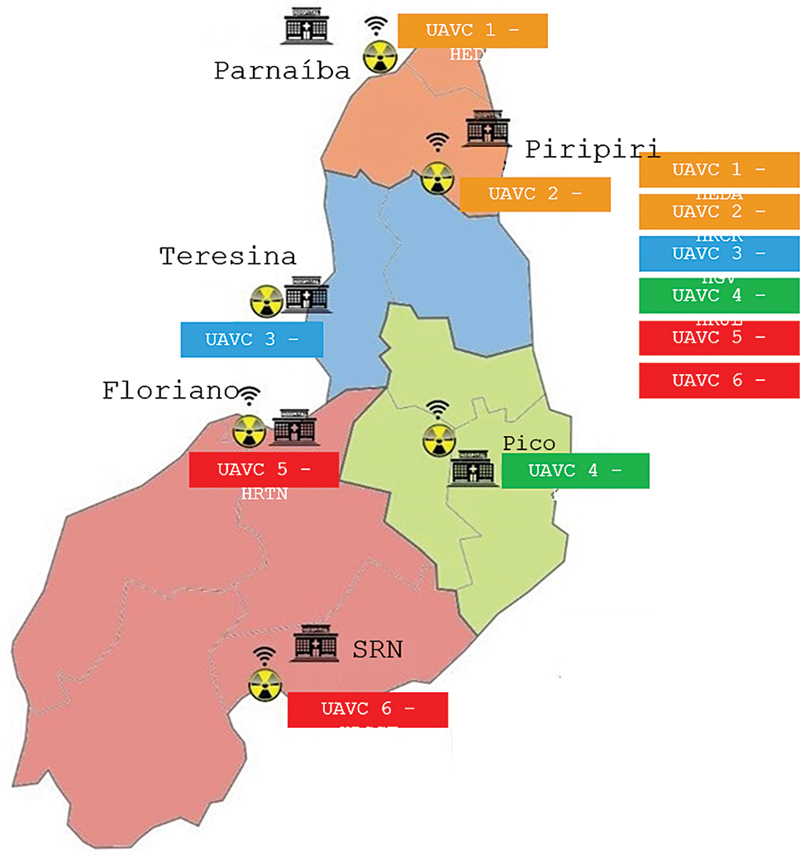
Abbreviations: HEDA, Hospital Estadual Dirceu Arcoverde; HRCR, Hospital Regional Chagas Rodrigues; HGV, Hospital Getúlio Vargas; HRJL, Hospital Regional Justino Luz; HRTN, Hospital Regional Tibério Nunes; HRSCF, Hospital Regional Senador Cândido Ferraz. Regionalization of Stroke Treatment Centers at regional hospitals in the state of Piauí.

The initiative to enhance stroke treatment in Piauí, Brazil, commenced in October 2022, with five Stroke Treatment Centers opened at regional hospitals in Floriano, Parnaíba, Picos, Piripiri, and São Raimundo Nonato. This initiative expanded in June 2023 when another center was inaugurated in Teresina, the state's capital ( Figure 1 ). Guided by the Ministry of Health's Ordinance 800, the selection of these centers was based on criteria emphasizing the availability of essential human and material resources. Initially, the five centers lacked specialized stroke units but offered thrombolytic treatment. The Teresina Center is distinguished by having both a stroke unit and the capability for mechanical thrombectomy. Plans are underway to establish five more centers to form an 11-center thrombolysis network across Piauí's macro-regions, all equipped for continuous tele-stroke support.

### Telemedicine and triage protocol

Medical teams contact a 24/7 telemedicine smartphone platform with 8 stroke neurologists for immediate imaging review and diagnosis. Patients within 4.5 hours of stroke onset are urgently referred to a nearby Stroke Reference Hospital. If onset exceeds 4.5 hours or if transportation issues arise, patients are sent to the nearest general hospital, possibly without specialized stroke evaluation. Uncertain or inconclusive cases by the Mobile Emergency Care Service (Serviço de Atendimento Móvel de Urgência, SAMU, in Portuguese) are also directed to general hospitals for further assessment. Reference hospitals, functioning as open-door facilities, also accommodate walk-in patients.

### Assessment and management strategy

At the stroke reference hospital, patients receive a thorough neurological evaluation and diagnostic imaging to verify the presence of an acute ischemic stroke. Upon confirmation, the treatment pathway can include intravenous thrombolysis or mechanical thrombectomy. The confirmation and management of stroke adhere to the protocols set forth by the Brazilian Stroke Society (Sociedade Brasileira de AVC, SBAVC, in Portuguese) and the Brazilian Academy of Neurology (Academia Brasileira de Neurologia, ABN, in Portuguese), 11 12 which are in line with international guidelines. 13

### Statistical analysis

Means, standard deviation, medians, and interquartile intervals were used to describe patients' characteristics. Categorical variables were presented as percentages. The Student's T and Mann-Whitney tests were used to compare quantitative variables for parametric and non-parametric samples, respectively. For paired groups, the T and Wilcoxon tests were used for parametric and non-parametric samples. Categorical variables were compared using the Chi-squared test. Statistical analysis was performed using Jamovi 2.3.28 statistical software.

## RESULTS

Our analysis, spanning from October 2022 to September 2023, incorporated data from 868 patients across 100 cities. The cohort's mean and median age were 67.2 and 69 years, respectively, with males accounting for 55.5% (462) of the cohort. There was no age difference between the 2 genders ( *p* > 0.05) ( Table 1 ).

**Table 1:** Epidemiological characteristics of stroke patients in Piauí (October 2022–September 2023)

Parameter		Value
Total of patients		868
Age: mean ± standard deviation (years)		67.2 ± 15.9
Age: median; 1st and 3rd quartiles (years)		69; 58 and 79
Male gender (%)		55.5%
Cities with the highest number of patients	Picos	105
	Parnaíba	86
	Teresina	62
	São Raimundo Nonato	54
	Floriano	52
	Piripiri	48
Preexisting comorbidities	Arterial hypertension (%)	57.8%
	Diabetes mellitus (%)	22.5%
Location of patient admission	Emergency room (%)	41.2%
	Admission to the Intensive care unit (%)	12.7%
	General ward (%)	16.0%
	Other hospital departments (%)	31.1%
	Stroke Unit hospitalization (%)	10.9%
Stroke diagnosis and treatment	Diagnosed with stroke or transient ischemic attack (%)	64.7%
	Ischemic stroke (%)	49.1%
	Hemorrhagic stroke (%)	8.2%
	Transient ischemic attack (%)	7.4%
Onset-to-door time (hours)		3.3 (CI: 3.0–3.5)
Door-to-computed tomography time (minutes)		23 (CI: 20.3–25.7)

The cities with the highest patient contributions were Picos (105), Parnaíba (86), Teresina (62), São Raimundo Nonato (54), Floriano (52), and Piripiri (48) ( Table 1 ).

Hypertension and diabetes mellitus (DM) emerged as the most common preexisting conditions among the participants, with 57.8% reporting a history of hypertension and 22.5% diagnosed with DM. There was no difference in the prevalence of hypertension between men and women ( *p* > 0.05, odds ratio [OR] 1.03 [CI 0.78–1.35]). However, the frequency of DM was higher in women ( *p* < 0.05, OR 0.69 [CI 0.50–0.95]) ( Table 1 ).

Hospitalization details indicated 41.2% of patients were admitted to the emergency room, 12.7% required Intensive Care Unit (ICU) care, 16% were in the general ward, and 31.1% were treated in other hospital departments ( Table 1 ).

A significant portion of the cohort, 64.7% (561 patients), was diagnosed with a stroke or TIA. Specifically, ischemic stroke was identified in 49.1% (425 patients), hemorrhagic stroke in 8.2% (71 patients), and TIA in 7.4%. The remainder of the group included stroke mimics or cases in which diagnosis was impeded by logistical or communication issues ( Figure 1 ). In our cohort, ischemic strokes accounted for 75.6% of cases, hemorrhagic strokes for 12.6%, and TIAs for 11.8% ( Table 1 ).

The mean onset-to-door time was recorded at 3.3 hours, and the door-to-CT time averaged 23 minutes. Thrombolysis with alteplase was administered to 135 patients, representing 31.8% of those with ischemic stroke (135/425) and an overall thrombolysis rate of 15.6% (135/868). Among patients who arrived within the thrombolytic time window, the rate was 27.3% (135/495). The average door-to-needle time was 67.1 minutes, and the mean NIHSS score was lower immediately postthrombolysis ( *p* < 0.05). Moreover, two patients underwent endovascular thrombectomy ( Table 2 ).

**Table 2:** Recanalization therapy parameters

Parameters	Value
Thrombolysis rate for ischemic stroke (%)	31.8 (135/425)
Thrombolysis rate in patients within 4.5 hours of onset (%)	27.3 (135/495)
Overall thrombolysis rate (%)	15.6 (135/868)
Mechanical thrombectomy (among ischemic stroke) (%)	0.5 (2/425)
Door-to-needle time (minutes)	67.1 (CI: 61.7–72.3)
NIHSS score before thrombolysis	14.2 (± 6.8)*
NIHSS score after thrombolysis	9.7 (± 7.6)*

Abbreviation: NIHSS, National Institute of Health Stroke Scale.

Note: * *p* < 0.05.

## DISCUSSION

In its first year, Piauí's stroke care initiative aligned with Brazil's national epidemiological trends 14 and pioneered the introduction of thrombolytic therapy in a previously unknown region. Showcasing exceptional efficiency, the program quickly transitioned patients from their arrival to diagnostic imaging, setting a precedent in patient care management. Despite these advancements, the time from arrival to treatment initiation has improved, but it still does not meet global stroke care guidelines recommendations. 13 This initiative achieved thrombolysis rates significantly above the national average, 15 16 17 establishing a new benchmark for stroke care in areas previously lacking access to these essential services.

In our analysis of the inaugural year of Piauí's integrated stroke care program, we observed that the epidemiological characteristics of stroke, including patient age, gender distribution, and the prevalence of risk factors, aligned closely with those reported in previous Brazilian studies. 14 15 17 Notably, our findings corroborate the pattern observed in other Brazilian cohorts, where the high frequency of intracerebral hemorrhages, commonly reported in South American stroke series, was not replicated. 14 15 17 18 19 20 Furthermore, the incidence of TIA in our study fell within the Brazilian prevalence range of 3 to 29% previously documented, 15 21 confirming the consistency of TIA occurrences across different regions. The rate of stroke mimics identified in our study, which includes patients presenting acutely with sudden onset neurological deficits, was comparable to that of prior research, showing a prevalence as high as 30% in some studies and ranging from 11 to 22% in telestroke settings. 7 Such findings emphasize the critical importance of accurate initial assessment and diagnosis in managing stroke and stroke-like episodes, underscoring the value of telestroke in extending specialist diagnostic support to remote areas.

Moreover, the distribution of patients from 100 cities, including major urban centers like Teresina and numerous rural locations, showcases the program's extensive reach and profound impact. This wide geographical spread of patients demonstrates the program's successful penetration across Piauí and highlights the effectiveness and necessity of its decentralized approach to stroke care. By facilitating access to specialist care and thrombolysis treatment across diverse settings, the program exemplifies a scalable model for stroke care delivery in regions with varied healthcare infrastructure.

Our program demonstrates commendable efficiency in patient management, with a mean onset-to-door time of 3.3 hours and a door-to-CT scan time of 23 minutes. These metrics reflect the program's capability to rapidly process patients from their arrival to diagnostic imaging. However, the average door-to-needle time of 67.1 minutes, although demonstrating significant progress in providing rapid care, still exceeds the international benchmarks recommended by stroke guidelines. 13 The critical nature of time in stroke treatment underscores the necessity of immediate intervention; the faster a patient receives thrombolytic therapy, the better the outcomes, 22 highlighting the importance of minimizing door-to-needle times. The importance of swift treatment is emphasized by stroke guidelines, advocating for the establishment of structured protocols and a multidisciplinary stroke team for the prompt evaluation of suspected stroke cases. In line with this, the SBAVC, 11 adhering to the American Stroke Association guidelines, 13 recommends conducting cranial CT scans within 25 minutes of arrival and initiating intravenous tissue-type plasminogen activator (tPA) therapy within 60 minutes for acute ischemic stroke patients.

In its 1 ^st^ year, our stroke care initiative in Piauí has made significant strides, achieving thrombolysis rates of 15.6% across all stroke patients and 31.8% among those with ischemic strokes. These figures are particularly noteworthy given the varied thrombolysis rates across Brazil—ranging from as low as 1.1% in the northeastern region to between 4.6 and 8.9% in the southeast, and 6 to 23% in the south. 15 17 21 23 The introduction of thrombolysis treatments in Piauí, a region previously without such services, has not only aligned with but also, in certain cases, exceeded the regional averages throughout the country. This achievement represents a major step forward in improving stroke care accessibility and quality in Brazil.

The notable success of our program is largely due to its focused strategy on identifying patients who are candidates for thrombolysis and improving the process for transferring those who fall outside the optimal treatment window. This method is in line with worldwide research showing the beneficial effects of telestroke services, which have led to a substantial increase in the use of thrombolytic therapy. Telestroke networks report thrombolysis rates ranging from 18 to 36%, far exceeding the 5 to 8% typically observed in the United States. 7 24 Such advancements are the result of targeted implementation and comprehensive training for telestroke services, as well as the strategic selection of patients based on local clinical guidelines. 25 For instance, the introduction of telestroke services in a network of hospitals increased thrombolysis rates from 2.8 to 6.8%, underscoring telestroke's profound capability to improve stroke treatment outcomes significantly.

Our study's findings, which show a reduction in NIHSS scores following thrombolysis, underscore the critical importance of timely intervention in stroke care. This aligns with existing research, highlighting thrombolytic therapy's effectiveness in improving acute ischemic stroke outcomes when administered within the crucial 4.5-hour window postsymptom onset. 26 This finding highlights the need for healthcare systems to enhance rapid response protocols for acute ischemic stroke, aiming to improve patient outcomes by ensuring early treatment.

Our retrospective study, conducted in hospitals in economically disadvantaged areas of Piauí, highlights significant challenges in analyzing stroke epidemiology due to inadequate record-keeping and incomplete data. The focus of our public stroke care program on thrombolysis-eligible patients, facilitated by telemedicine, may introduce selection bias, as this subgroup does not fully represent the broader stroke patient population in the region. Addressing these limitations is crucial for accurate interpretation of our findings.

To mitigate these challenges, we are implementing standardized data collection protocols and enhancing training for data collectors across participating institutions. These efforts aim to improve data accuracy and comprehensiveness, enabling more robust analyses of stroke care outcomes in Piauí. Further research is also needed to guide program improvements and provide a more comprehensive understanding of stroke care.

While thrombolysis rates in our program are above the national average, achieving further improvements is hindered by regional disparities in healthcare infrastructure, access to trained professionals, and availability of stroke units. These challenges are particularly pronounced in economically disadvantaged areas, where resources are limited and reliance on telemedicine is greater. Addressing these barriers will require targeted investments in infrastructure, workforce training, and equitable resource allocation.

To address these disparities, we are expanding our stroke care infrastructure, aiming to complete this initiative by 2026. This includes establishing five additional specialized stroke units and extending thrombolysis coverage to underserved regions. Plans are also underway to develop five more centers, creating an 11-center thrombolysis network across Piauí's macro-regions, all supported by tele-stroke technology.

As of 2024, significant progress has been made. A new thrombolysis center was established in the city of Campo Maior, and in December 2024, the state's second stroke unit was inaugurated in Floriano. By the first half of 2025, we plan to open additional stroke units in the cities of Picos and Parnaíba. These developments aim to improve patient management, expand access to life-saving treatments, and enhance data collection on patient outcomes, thrombolysis safety, and epidemiological trends. This initiative reflects our commitment to advancing stroke care services, ensuring equitable access to high-quality treatment for all patients in Piauí, and emphasizes the importance of ongoing research and development in this field.

The achievements of our integrated care program, powered by telemedicine, represent a crucial step forward in mitigating healthcare disparities and improving stroke care in Piauí. This progress not only illustrates the program's potential as a blueprint for similar regions globally but also underscores the need for continued enhancements in healthcare infrastructure and professional training.
